# Use of Whey Protein as a Natural Polymer for Tissue Adhesive: Preliminary Formulation and Evaluation In Vitro

**DOI:** 10.3390/polym10080843

**Published:** 2018-07-30

**Authors:** Guorong Wang, Ning Liu, Mingruo Guo

**Affiliations:** 1 Department of Foods Science, The Northeast Agricultural University, Harbin 150030, China; guorong_wang@yahoo.com; 2 Department of Nutrition and Foods Sciences, The University of Vermont, Burlington, VT 05405, USA; nliu2224@163.com

**Keywords:** whey protein, surgical glue, crosslink, bonding strength, non-food

## Abstract

The use of sutures is still the most widely practiced solution for wound closure and tissue reconstruction; however, scarring is a common defect resulting from sutures on topical use. In some cases, the conventional sutures are unable to seal the sites where fluid and air leakage could occur. Tissue adhesives though have lower tensile strength than sutures, may make scarless surgery possible, or prevent fluid and air leakage. A product called BioGlue^®^ (CryoLife Inc, Kennesaw, GA, USA), based on bovine serum albumin (BSA, a protein) and glutaraldehyde (GTA, crosslinker), has been approved for clinical use in the USA. Whey protein, a byproduct of cheese-making, comprised mainly of β-lactoglobulin, α-lactalbumin and BSA. Even though the molecular weight of BSA is about three times larger than the molecular of β-lactoglobulin and α-lactalbumin, all three proteins are rich in free ε-amino groups (can react with GTA) and globular proteins. This similarity make whey protein a potential candidate to replace BSA in the tissue adhesive since whey protein is abundant and much cheaper than BSA. In this study, whey protein isolate (WPI) was used as a protein polymer with GTA as a crosslinker to evaluate the feasibility of whey protein for tissue adhesive formulation. Results showed that the WPI/GTA adhesive exhibited a comparable adhesive strength to BioGlue^®^ control.

## 1. Introduction

Sutures, staples, clips, and surgical tapes are the common techniques for wound closure and tissue reconstruction. The primitive application of sutures can be dated back to ancient times [1]. To date, the sutures are still considered the optimal practice for closing wound or surgery incisions. Both resorbable and non-resorbable materials are available for specific applications [1,2,3]. However, the disadvantages of mechanical sutures are also obvious, like physical pain, scarring, prolonged learning curve for practitioners, and the inability to prevent fluid or gas leakage [2,4,5]. Tissue adhesives or surgical adhesives, a new alternate to sutures, have been developed since the 1950s and especially after the 1980s when the first surgical adhesive was approved for clinical use in Europe and Canada [1]. The use of tissue adhesive could reduce the physical pain, prevent fluid leakage, and shorten operation time [1,6,7].

**Figure 1:**
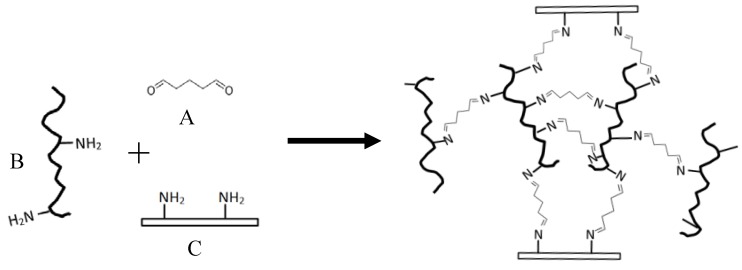
Reaction mechanism of protein polymer and GTA adhesive to bond tissue cells. (A) GTA; (B) protein polymer molecules; and (C) tissue or organ proteins.

Tissue adhesives can be categorized into biological (e.g., fibrin sealant), composite biological (e.g., on the basis of albumin and glutaraldehyde (GTA)), and synthetic (e.g., cynaoacrylate adhesive) [1]. A composite biological tissue adhesive usually contains two components, i.e., protein polymer and crosslinker. The albumin/GTA surgical adhesive branded under the name BioGlue^®^ is approved for clinical use by the Food and Drug Administration (FDA). Extensive in vivo evaluations of BioGlue^®^ have been published during past decades [8,9,10,11,12,13,14]. The GTA with its two carbonyl groups can crosslink the adhesive protein as well as with the tissue proteins in both cases via the reaction with the free amino groups of the various proteins (Figure 1) [1]. The free amino groups in the adhesive protein are mainly the ε-NH_2_ of lysyl residue, which is rich in BSA.

Purified BSA is very expensive and access to obtain it may be limited; while purified whey protein is a much cheaper with similar functionality to BSA. Whey protein is isolated from whey—a byproduct of cheese-making [15]. Liquid whey directly separated from cheese preparation only contains about 0.6–0.7% protein; the majority in liquid whey is lactose (4–5%) [16]. Advanced protein purification technologies, such as membrane filtration, ion-exchange, electrodialysis, and chromatography, are capable to produce a very pure whey protein, such as whey protein isolate (WPI) with a protein content over 90% [17,18,19]. Whey protein mainly comprise β-lactoglobulin (45–55%), α-lactalbumin (20–25%), and BSA (5–10%) [20,21,22]. WPI has excellent functionalities due to its high content of protein (higher than 90%). It is currently widely used in various food and nonfood applications, such as protein supplements, emulsifiers, adhesives, and coatings [23,24,25,26,27]. 

Whey protein contains a significant level of BSA, which is originally from blood. During milk synthesis, BSA, the albumin present in bovine blood, can directly permeate the cell membrane becoming part of milk components [28]. BSA and other major whey proteins (β-lactoglobulin and α-lactalbumin) are all globular proteins which are soluble in acid and resistant to rennet (the common yogurt—and cheese—making process), thus can be separated from yogurt or cheese curd. The free ε-amino groups in BSA form strong chemical bonds with GTA. The reaction mechanism was depicted in Figure 1. The numbers of ε-amino groups are 37, 27, and 113 out of 162, 125, and 583 of the total amino acid residues of β-lactoglobulin, α-lactalbumin, and BSA, respectively [29,30,31], which provide theoretical maximum of one crosslink per 4.38, 4.63, and 5.16 amino acid residues (or per 494, 525, and 588 Dalton molecular weight) for β-lactoglobulin, α-lactalbumin, and BSA, respectively. 

Therefore, we hypothesize that whey protein could be an alternate polymer candidate for biological adhesive because it contains a group of globular proteins including BSA and has as many free amino groups as BSA. More importantly, whey protein is more abundant and economical than isolated BSA. The objectives of this study were to formulate a whey protein-based tissue adhesive and to evaluate its adhesive properties (lap-shear bonding strength, bonding time, and wound closure strength). The commercial BSA/GTA BioGlue^®^ was used as a comparison. 

## 2. Materials and Methods

### 2.1. Glue Components Preparation

The adhesive mix comprised two components, the WPI solution and the GTA solution. WPI (protein 90.4%, and total solids 95.58%) was purchased from Fonterra Ltd. (Auckland, New Zealand). At refrigerated temperature, the WPI was rehydrated in sterilized deionized water (4 °C) at a concentration of 4%, and then filtered (4 °C) by a Millex™ syringe with sterile filter unit (pore size: 0.22 µm) (Millipore Corporation, Bedford, MA, USA) to remove bacterial cells. The filtrate was freeze dried by a FreeZone^®^ 4.5 Liter Freeze Dry Systems (model 7751020) (Labconco Corporation, Kansas, MO, USA). The whole process was conducted under sterile conditions to minimize contamination. The sterilized freeze-dried WPI powder was reconstituted using sterilized deionized water to make the WPI solution at concentrations of 30.0%, 35.0%, 40.0%, and 45.0% (*w*/*w*). GTA (50.0% solution, *w*/*w*) purchased from Fisher Scientific (Fair Lawn, NJ, USA) was diluted using sterilized deionized water to 6.0%, 8.0%, 10.0%, and 12.0% (*w*/*w*), respectively. 

### 2.2. Gelation Time

The gelation time of the WPI/GTA mix was determined as described by Mo and Iwata et al. (2010). The WPI solution (4.00 mL) was added in a clear glass tube (10 mL of total volume) and placed on a stir plate at ambient temperature, and stirred with a magnetic micro stir bar (diameter 3 mm and length 10 mm) (Fisherbrand™, Ottawa, ON, Canada) at 50 rpm. When 1 mL of GTA solution was added using a pipette, start timing. The mixture would gel quickly, when the gel became hardened and the micro stir bar was stuck in the gel, timing was stopped. The time required for the stir bar to stop stirring was recorded as gelation time [32].

### 2.3. Lap-Shear Bonding Strength

**Figure 2:**
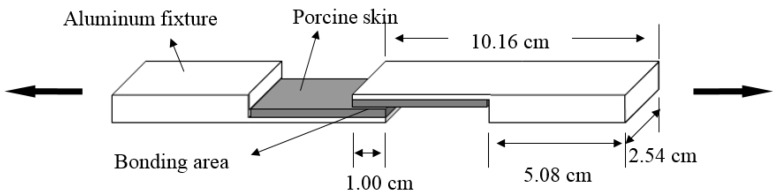
Depiction of lap-shear bonding strength testing apparatus.

Lap-shear bonding strength was tested according to the method of ASTM F2255-05 [33]. Fresh porcine skin was purchased from a local market (South Burlington, VT, USA). The skin graft was cut into the dimensions of 5.08 cm × 2.54 cm × 0.24 cm with a #20 Uniblade^TM^ disposable surgical scalpel (AD Surgical, Sunnyvale, CA, USA), The skin strip was glued on an aluminum block with dermal side up by using a Loctite^®^ super glue (Henkel Corporation, Rocky Hill, CT, USA). The test specimens were kept moist by being wrapped in gauzes soaked with phosphate-buffered saline (PBS) (Fisher Scientific, Fair Lawn, NJ, USA) and placed in an environmental chamber at 23 °C and 50% relative humidity. A total of 100 µL of WPI solution and 25 µL of GTA solution was applied to the skin’s dermal side, mixed with a small steel spatula, and then lapped with another porcine skin strip backed by the fixture as described in Figure 2. The bonding area was 2.54 cm × 1.0 cm. The newly glued specimens were being clamped by Staples^®^ #4 Bulldog clips (purchased from Staples, South Burlington, VT, USA) and conditioned at 23 °C for 30 min being wrapped by PBS soaked gauzes. The lap-shear bonding strength was tested by an Instron 5566 universal testing machine (Instron Corporation, Canton, MA, USA). The glued specimens were placed in the grips (maximum load: ±500 N) of the Instron testing machine and operated at a rate of 10.00 mm/min until the test specimens ripped apart. The maximum load (N) was recorded and lap-shear bonding strength (kPa) was calculated by dividing the maximum load (N) by the bonding area 1.00 cm × 2.54 cm).

### 2.4. Bonding Curve

The lap-shear bonding strength of joints prepared by the WPI/GTA adhesive were tested after conditioning at 23 °C for different periods of time (from 1 to 30 min). The bonding strength was plotted vs. the conditioning time. The time to achieve the maximum bonding strength was marked as the bonding time.

### 2.5. Wound Closure Strength

**Figure 3:**
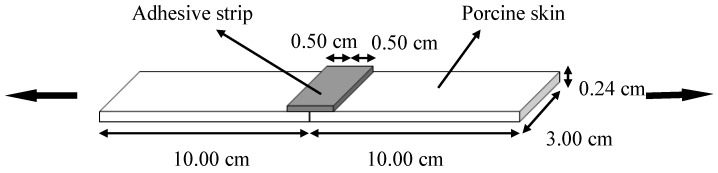
Depiction of wound closure strength test specimen.

**Figure 4:**
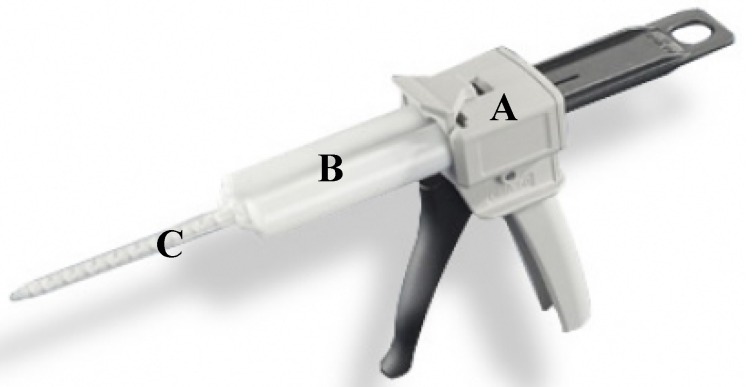
Mixpac mixing device. (A) Dispensing gun; (B) dual barrel cartridge system (4:1); and (C) static mixer.

Wound closure strength was tested by a 5566 testing machine (Instron Corporation, Canton, MA, USA) according to the method of ASTM F2458-05 [32]. Fresh porcine skin was cut into dimensions of 10.00 cm × 3.00 cm × 0.24 cm. Two porcine skin strips were lined up, as depicted in Figure 3. The WPI/GTA adhesive was prepared by a Mixpac^®^ mixing device (Figure 4, AP Plastics, LLC., Peabody, MA, USA), with dispensing gun (DS 51 4:1), a dual barrel cartridge system (50 mL, AS 050-04-09-1X), and a static mixer (MBHX 04-16S). The cartridge system had two barrels with prefixed ratio of 4:1. An aluminum template was placed at the connection area of the two skin strips and the WPI/GTA adhesive was applied there to form an adhesive strip with dimension of 1.00 cm × 3.00 cm × 0.24 cm as described in Figure 3. The test specimen was kept moist all the time by being wrapped in PBS soaked gauzes. After being conditioned at 23 °C for 30 min, the glued specimen was clamped by the Instron grips and operated at a speed of 10 mm/min. The peak load (N) at failure was recorded as the wound closure strength. 

### 2.6. Statistical Analysis

Lap-shear bonding strengths and wound closure strengths were the averages of 10 valid tests; other data were the averages of three tests. One-way ANOVA analysis was conducted at a significant level of 95.0% by SPSS 16.0 software (SPSS Inc., Chicago, IL, USA).

## 3. Results and Discussion

### 3.1. Effects of Concentrations of WPI and GTA on the Lap-Shear Bonding Strength

**Figure 5:**
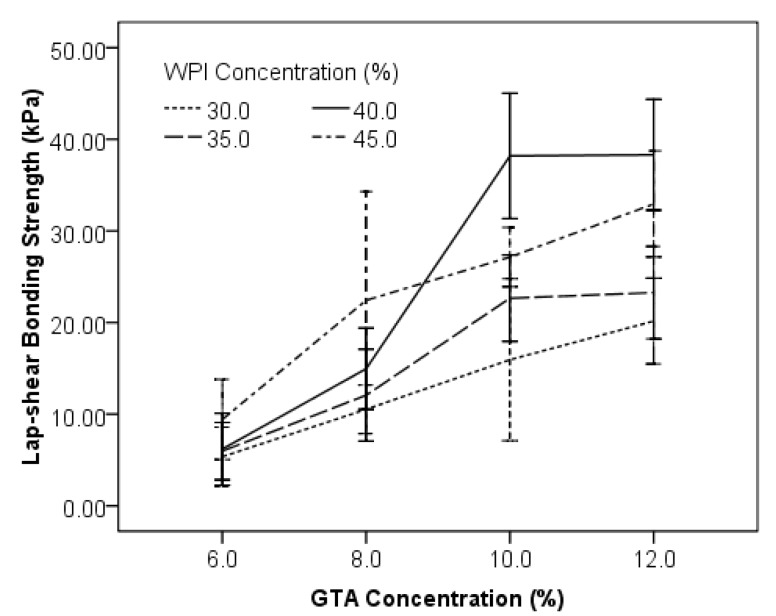
Effects of WPI and GTA concentrations on lap-shear bonding strength.

WPI has excellent solubility in water and can form high content homogenous solutions up to 50%. WPI solutions with different concentrations (30.0%, 35.0%, 40.0%, and 45.0%) were mixed with GTA solutions of different concentration (6.0%, 8.0%, 10.0%, and 12.0%) at a ratio of 4:1 in volume. The lap-shear bonding strength of those combinations was evaluated. Results indicated that higher concentrations of both WPI and GTA levels had positive effects on lap-shear bonding strength (Figure 5). When GTA was 6.0%, the lap-shear bonding strength for protein content at 30.0% (5.23 ± 3.26 kPa), 35.0% (5.32 ± 3.78 kPa) and 40.0% (5.33 ± 3.54 kPa) of WPI were almost identical, but lower than the sample composed of 45.0% WPI (10.05 ± 4.65 kPa). Higher levels of WPI resulted in higher bonding strength at GTA level of 8.0% (Figure 5). However, when GTA was 10.0% or 12.0%, the bonding strength of 45.0% WPI was lower than that of 40.0% WPI, and no further increase of bonding strength was found for 40.0% WPI when GTA was increased from 10.0% to 12.0%. The combinations of WPI (40.0%)/GTA (10.0%) and WPI (40.0%)/GTA (12.0%) had bonding strength of 38.2 ± 9.6 kPa and 38.7 ± 7.9 kPa, respectively, which were comparable (*p* > 0.05) to the BioGlue^®^ (40.1 ± 12.2 kPa).

In general, a higher concentration of WPI or GTA resulted in higher bonding strength, based on the higher concentration introduced higher numbers of ε-NH_2_ groups (protein) and carbonyl groups (GTA), which are the two functional groups that crosslinked to form the bonding strength. WPI of 40% has the highest bonding strength compared to other concentrations. Viscosity or flowability that impact the applicability of the adhesive may play an important role [23]. WPI solutions at 30% and 35% are less viscous and flowable, whose viscosity are 28.8 ± 2.9 mPa·s and 48.1 ± 1.1 mPa·s, respectively. When these two lower concentrated WPI were applied to the pork skin, immediate seeping occurred which could cause the low bonding strength due to less adhesive remaining in the bond line. The 40% WPI solution has a 415 ± 11 mPa·s viscosity and very easy to apply and to mix; the 45% WPI was too viscous (viscosity of 2203 ± 23 mPa·s) to be spread, and it also required additional pressure to be driven in the Mixpac syringe device. 

### 3.2. Effects of GTA Concentration on Wound Closure Strength

**Figure 6:**
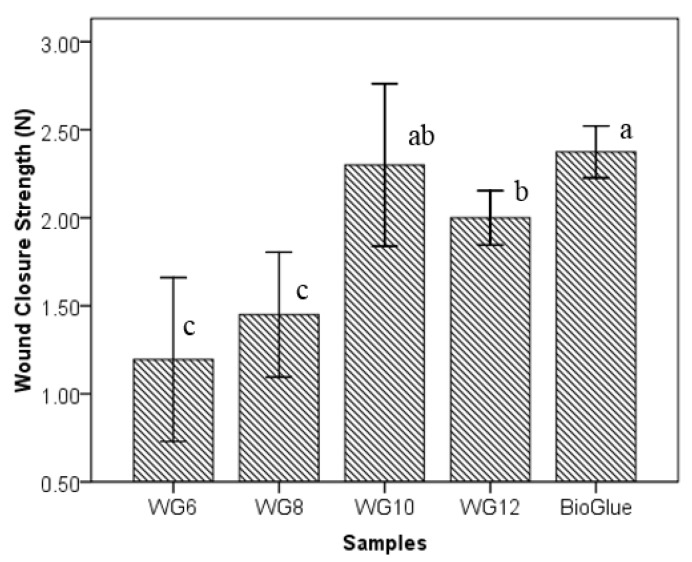
Effects of GTA on wound closure strength of WPI/GTA adhesive (The annotated letters indicate the statistical difference at significant level of 95.0%).

The wound closure strength is mimicking the adhesive functions as a topical band to hold the wound edges together and the wound closed, thus shortening the wound healing time [34]. The 40% WPI solution was mixed with 6.0%, 8.0%, 10.0%, and 12.0% GTA solutions, respectively, at a volume ratio of 4:1 to evaluate the gelation time. The samples were recoded as WG6 (40% WPI + 6.0% GTA), WG8 (40% WPI + 8.0% GTA), WG10 (40% WPI + 10.0% GTA), and WG12 (40% WPI + 10.0% GTA). The wound closure strength of WG6, WG8, WG10, and WG12 were 1.2 ± 0.5 N, 1.5 ± 0.4 N, 2.3 ± 0.5 N and 2.0 ± 0.2 N, compared with 2.4 ± 0.2 N of BioGlue^®^ (Figure 6). The closure strength of WG6 and WG8 were significantly lower (*p* < 0.05) than WG10, WG12, and BioGlue^®^. The mean value of WG12 was lower than WG10, but no significance difference was detected (*p* > 0.05). WG10 had a comparable (*p* > 0.05) bonding strength to BioGlue^®^ (2.30 ± 0.46 N versus 2.37 ± 0.15 N). The effects of the amount of GTA on the lap-shear strength and the wound closure strength are similar. The two types of strength both reached the peak at a GTA concentration of 10.0%. A further increase in GTA concentration did not improve the bonding strength.

### 3.3. Effects of GTA Concentration on Gelation Time

**Figure 7:**
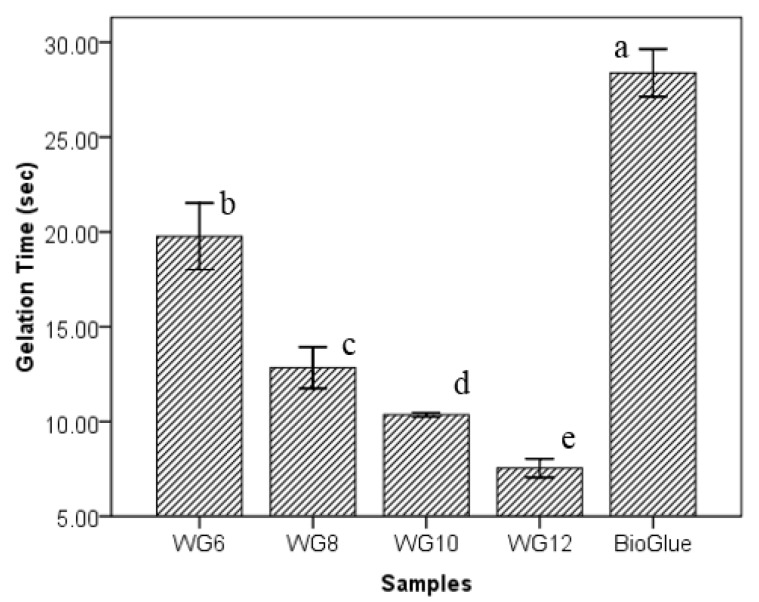
Gelation time of WPI/GTA adhesives and BioGlue^®^ (WG6, WG8, WG10, and WG12 were 40.0% of WPI solutions mixed with 6.0%, 8.0%, 10.0%, and 12.0% of GTA solutions at a ratio of 4:1 in volume) (The annotated letters indicate the statistical difference at significant level of 95.0%).

The two components of the adhesive (40% WPI solution was mixed with 6.0%, 8.0%, 10.0%, and 12.0% of GTA solutions; at a volume ratio of 4:1) mixed in a tube congealed quickly due to crosslinking between the protein and the GTA molecules. A higher concentration of GTA shortened the gelation time significantly (Figure 7) due to the higher available amount of carbonyl groups at higher GTA concentration. The gelation times of WPI/GTA adhesives were 19.8 ± 1.8 s (WG6), 12.8 ± 1.1 s (WG8), 10.4 ± 0.1 s (WG10) and 7.5 ± 0.5 (WG12), compared to the BioGlue^®^ control (28.4 ± 1.3 s).

The gelation time is the indicator how long the adhesive mix needs to cure and to create cohesive adhesive strength after it is applied. If the gelation time is too short, the operators may not have enough time to do any readjustment, or even the glue cures before lining up the adherend. On the other hand, if gelation time is too long, it requires extended time of holding the adherend in place before the adhesive is set. An appropriate gelation time, therefore, is very important for a surgical adhesive.

### 3.4. Bonding Curves of the WPI/GTA Glue

**Figure 8:**
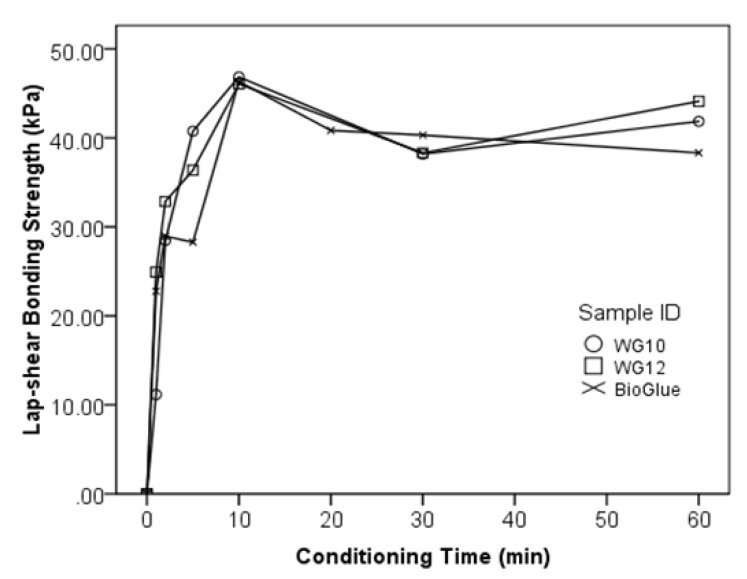
Relationship between lap-shear bonding strength and conditioning time after application of WPI/GTA adhesive and BioGlue^®^.

The bonding curves indicate the increase of bonding strength vs. time after the adhesive mix is applied, and when the maximum bonding strength is obtained. The bonding curves of WG10, WG12, and BioGlue^®^ are shown in Figure 8. WG10 and WG12 exhibited 11.2 kPa and 24.9 kPa of lap-shear bonding strength already 1 min after application and reached the peak of bonding strength at 10 min; there was more or less no difference in the curves between WG10, WG12 and BioGlue^®^: the lower value at 5 min for the BioGlue^®^ seems to be influenced by other parameters rather than by the curing reaction. The difference in gelation time between the two WG versions and the BioGlue^®^ were not confirmed by the bonding curves. The lap-shear bonding strengths increased at high speed during the first 10 min and then remained stable; this shows that it takes about 10 min for the adhesive mixes investigated to reach their maximum strength. 

## 4. Conclusions

The whey protein/GTA system has comparable bonding strength (both lap shear and wound closure) compared to the BSA control BioGlue^®^. The results indicated that whey protein would be a good alternative protein polymer to BSA for tissue adhesive formulation, in terms of the adhesive strength. Whey protein is considered as a waste material from cheese making is much cheaper than BSA. In order to develop a practical tissue adhesive, a medical grade of the protein source is needed. Further in vitro and in vivo evaluations are needed to further understanding the feasibility of whey protein in this application.
